# Regulation of AMPA Receptor Trafficking by Protein Ubiquitination

**DOI:** 10.3389/fnmol.2017.00347

**Published:** 2017-10-26

**Authors:** Jocelyn Widagdo, Sumasri Guntupalli, Se E. Jang, Victor Anggono

**Affiliations:** Clem Jones Centre for Ageing Dementia Research, Queensland Brain Institute, The University of Queensland, Brisbane, QLD, Australia

**Keywords:** AMPA receptors, ubiquitin, E3 ligase, deubiquitinating enzyme (DUB), endosomal sorting, lysosome, protein degradation, synaptic plasticity

## Abstract

The molecular mechanisms underlying plastic changes in the strength and connectivity of excitatory synapses have been studied extensively for the past few decades and remain the most attractive cellular models of learning and memory. One of the major mechanisms that regulate synaptic plasticity is the dynamic adjustment of the α-amino-3-hydroxy-5-methyl-4-isoxazolepropionic acid (AMPA)-type glutamate receptor content on the neuronal plasma membrane. The expression of surface AMPA receptors (AMPARs) is controlled by the delicate balance between the biosynthesis, dendritic transport, exocytosis, endocytosis, recycling and degradation of the receptors. These processes are dynamically regulated by AMPAR interacting proteins as well as by various post-translational modifications that occur on their cytoplasmic domains. In the last few years, protein ubiquitination has emerged as a major regulator of AMPAR intracellular trafficking. Dysregulation of AMPAR ubiquitination has also been implicated in the pathophysiology of Alzheimer’s disease. Here we review recent advances in the field and provide insights into the role of protein ubiquitination in regulating AMPAR membrane trafficking and function. We also discuss how aberrant ubiquitination of AMPARs contributes to the pathogenesis of various neurological disorders, including Alzheimer’s disease, chronic stress and epilepsy.

## Introduction

The binding of glutamate to α-amino-3-hydroxy-5-methyl-4-isoxazolepropionic acid (AMPA) receptors (AMPARs) mediates the fast, moment-to-moment transmission of excitatory signals in the mammalian central nervous system. The mammalian genome encodes four AMPAR subunits, GluA1–4. These combine as a dimer of dimers to form functional tetramers that are generally permeable only to Na^+^ and K^+^ ions, with the exception of GluA2-lacking AMPARs, which also conduct additional Ca^2+^ ions into dendritic spines (Sukumaran et al., 2012). AMPARs cycle into and out of the neuronal plasma membrane under basal conditions, and these trafficking patterns can be rapidly and dynamically modulated in an activity-dependent manner. Dynamic trafficking of AMPARs is one of the major mechanisms underpinning various forms of synaptic plasticity, including Hebbian and homeostatic plasticity (Pozo and Goda, 2010; Huganir and Nicoll, 2013). These processes are tightly regulated by the orchestrated binding of AMPAR binding proteins, as well as by reversible post-translational modifications that occur on the carboxyl terminal domains of AMPAR subunits (Anggono and Huganir, 2012; Lu and Roche, 2012).

Ubiquitination is a reversible post-translational modification that regulates a myriad of physiological processes, including protein degradation, endocytosis and the sorting and trafficking of transmembrane proteins (Hershko and Ciechanover, 1998). It involves the covalent attachment of a highly conserved 76 amino acid ubiquitin moiety (monoubiquitination) or polymeric ubiquitin chains (polyubiquitination) to a lysine residue of a substrate protein (Hershko and Ciechanover, 1998). The conjugation of ubiquitin to a substrate depends on an enzymatic cascade that comprises ubiquitin-activating enzymes (E1s), ubiquitin-conjugating enzymes (E2s) and ubiquitin ligases (E3s). Although the ubiquitin pathway is important in all cells, it is now clear that this pathway subserves a range of important functions in neurons and is necessary for learning and memory (Mabb and Ehlers, 2010). Importantly, the ubiquitin-proteasome system is crucial in regulating AMPAR trafficking and turnover (Patrick et al., 2003; Zhang et al., 2009; Yuen et al., 2012). The evidence demonstrating the ubiquitination of the glutamate receptor, GLR-1 and its role in regulating receptor abundance was first obtained in the nematode *C. elegans* (Burbea et al., 2002). Since then, several studies have also reported the ubiquitination of AMPARs in mammalian neurons (Schwarz et al., 2010; Fu et al., 2011; Lin et al., 2011; Lussier et al., 2011; Widagdo et al., 2015). In this review, we highlight recent progress in the field that advances our understanding of the molecular regulation of AMPAR function by protein ubiquitination and its potential implication in the treatment of various disorders, including Alzheimer’s disease, epilepsy and chronic stress. Given the limited knowledge on the regulation of the GluA3 and GluA4 subunits of AMPARs by post-translational ubiquitination, this review focuses only on GluA1 and GluA2, the heteromers of which constitute the majority of AMPARs in the forebrain.

## Mechanisms Underlying the Ubiquitination of AMPARs

### Ubiquitination of AMPARs Is Ca^2+^-Dependent

**Figure 1:**
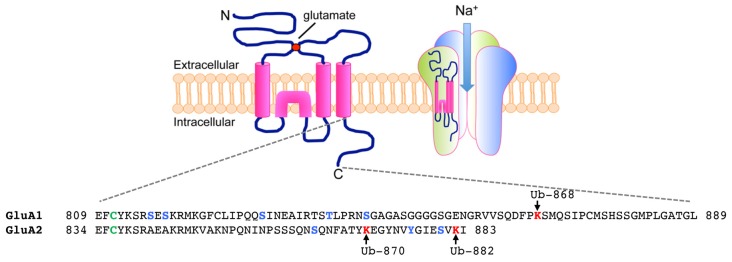
Ubiquitination of α-amino-3-hydroxy-5-methyl-4-isoxazolepropionic acid receptors (AMPARs) on the C-terminal of GluA1 and GluA2 subunits. (Top) Each AMPAR subunit is composed of an extracellular glutamate binding region, four transmembrane domains, two intracellular loops and an intracellular carboxy-tail. Four subunits (two homodimers) are assembled into a functional tetramer that is permeable to Na^+^ ions. (Bottom) Amino acid sequences of the carboxy-tails of GluA1 and GluA2 showing sites of post-translational ubiquitination (lysines in red, arrows), phosphorylation (serines, threonines and tyrosine in blue) and palmitoylation (cysteines in green).

The ubiquitination of AMPARs is initially triggered by the binding of ligand to the receptors. All four subunits of AMPARs, GluA1-4, undergo activity-dependent ubiquitination when neurons are stimulated with AMPA or bicuculline (Schwarz et al., 2010; Lussier et al., 2011; Widagdo et al., 2015). As a selective GABA_A_ receptor antagonist, bicuculline enhances the release of glutamate from the presynaptic terminals and therefore preferentially activates AMPARs that are located at the synapse, while bath application of AMPA co-activates both synaptic and extrasynaptic receptors. The major ubiquitination site for the GluA1 subunit has been mapped to Lys-868 located in the distal C-terminal tail of the receptor, whereas GluA2 is predominantly ubiquitinated at Lys-870 and Lys-882 in neurons (Figure 1; Widagdo et al., 2015).

In addition to ligand binding to AMPARs, subsequent depolarization of the postsynaptic membrane is also required for AMPAR ubiquitination. This allows the second messenger Ca^2+^ to enter the postsynaptic compartment through L-type voltage-gated Ca^2+^ channels (L-VGCCs) and activates a Ca^2+^-dependent signaling cascade that involves the activation of Ca^2+^/calmodulin-dependent kinase II (CaMKII; Lussier et al., 2011; Widagdo et al., 2015). Mechanistically, the role of CaMKII in regulating AMPAR ubiquitination is currently unknown, but CaMKII is presumably involved in direct phosphorylation and/or activation of E3 ligase(s) for AMPAR subunits. Interestingly, neither agonist-induced activation of NMDA receptors (NMDARs) nor NMDAR-dependent signaling is required for AMPA-induced ubiquitination of AMPARs (Schwarz et al., 2010; Lussier et al., 2011; Widagdo et al., 2015). However, the activity of NMDARs plays an important role in modulating the ubiquitination of AMPARs induced by bicuculline (Lussier et al., 2011; Widagdo et al., 2015). These findings suggest that AMPA and bicuculline stimulate two molecularly distinct signaling pathways, which may result in the recruitment of different E3 ligases and dictate the routes of AMPAR trafficking and subsequent degradation through the lysosomal or proteasomal pathways.

### E3 Ligases for AMPARs

To date, four different E3 ligases, namely neural precursor cell-expressed developmentally downregulated gene 4-1 (Nedd4-1), Nedd4-2, RNF167 and APC^Cdh1^, have been shown to mediate the ubiquitination of AMPARs in mammalian central neurons under different stimulation conditions (Schwarz et al., 2010; Fu et al., 2011; Lin et al., 2011; Lussier et al., 2012; Zhu et al., 2017).

#### Nedd4

The Nedd4 family of E3 ligases is characterized by the presence of the homologous to E6-AP C-terminus (HECT) domain, which first accepts ubiquitin from an E2 ligase onto its catalytic cysteine residue, prior to transferring it to the substrate (Scheffner and Kumar, 2014). The human genome encodes nine members of the Nedd4 E3 ligase family, each of which contains an N-terminal C2 Ca^2+^/phospholipid-binding domain, multiple WW protein-protein interaction domains and a C-terminal HECT domain. These ligases preferentially form K63-linked polyubiquitin chains on their substrates (Kim et al., 2007). Nedd4-1 was the first E3 ligase which was shown to interact with and facilitate the ubiquitination of GluA1 in neurons (Schwarz et al., 2010). Overexpression of Nedd4-1 reduces surface AMPARs due to enhanced endocytosis and the accumulation of internalized GluA1 in late endosomes (Schwarz et al., 2010; Lin et al., 2011; Scudder et al., 2014). Interestingly, Nedd4-1 is rapidly redistributed to synapses upon AMPAR activation (Hou et al., 2011; Scudder et al., 2014). This activity-dependent localization of Nedd4-1 requires the functional C2 domain (Scudder et al., 2014), presumably to mediate Ca^2+^-dependent binding to phospholipids (Plant et al., 1997). Furthermore, overexpression of Nedd4-1 with deletion of the C2 domain fails to cause any reduction in synaptic AMPARs, suggesting the importance of Nedd4-1 Ca^2+^-binding in regulating GluA1 surface expression (Scudder et al., 2014).

More recently, another closely related E3 ligase, Nedd4-2 (also known as Nedd4-like) has also been demonstrated to facilitate GluA1 ubiquitination in neurons (Jewett et al., 2015; Zhu et al., 2017). Nedd4-2, when bound to the adaptor protein 14-3-3ε, can directly ubiquitinate GluA1 in an *in vitro* ubiquitination assay (Zhu et al., 2017). Loss of Nedd4-2 function inhibits picrotoxin-induced ubiquitination of GluA1 in primary neurons (Jewett et al., 2015). As a consequence, the expression of GluA1 is elevated in the brain of the seizure prone Nedd4-2^andi^ mouse, in which the major isoform of Nedd4-2 is deleted (Zhu et al., 2017).

Although Nedd4-1 and Nedd4-2 are structurally very similar, they appear to have distinct cellular functions and target specific proteins for ubiquitination (Yang and Kumar, 2010). Many of the identified Nedd4-1 substrates are receptor tyrosine kinases, whereas Nedd4-2 preferentially targets ion channels (Persaud et al., 2009). However, Nedd4-1 and Nedd4-2 also share several common substrates, which are likely to include GluA1 in the brain. It is plausible that the recruitment of these two E3 ligases might be regulated by different types of neuronal stimulation, providing selectivity and specificity in the regulation of AMPAR trafficking and function.

#### RNF167

RING finger protein (RNF) 167 is a member of the really interesting new gene (RING) E3 ligase family that comprises over 600 proteins in the human genome. Unlike the HECT domain, the RING domain lacks a catalytic cysteine and merely acts as a scaffold to facilitate the direct transfer of ubiquitin from E2 to the substrate. RNF167 is an integral membrane protein that is localized to the plasma membrane, endosomes and lysosomes, and has a role in regulating endosomal trafficking and the degradation of substrates in lysosomes (Lussier et al., 2012; Yamazaki et al., 2013; van Dijk et al., 2014; Deshar et al., 2016). It was first identified as an E3 ligase for the GluA2 subunit of AMPARs (Lussier et al., 2012). shRNA-mediated knockdown of RNF167 significantly reduces GluA2/3 ubiquitination in neurons following bicuculline stimulation. Interestingly, loss of RNF167 function not only enhances the expression of surface GluA2/3, but also upregulates surface GluA1 levels in cortical neurons. This suggests that RNF167 could be another E3 ligase for GluA1, although this has not been demonstrated biochemically. In addition, whether RNF167 is specifically recruited during heightened synaptic activity to ubiqutinate only the GluA2 and GluA3 subunits is yet to be determined.

#### APC^Cdh1^

Anaphase promoting complex (APC) is a large multisubunit E3 ligase that targets key cell cycle regulatory proteins for proteasomal degradation (Huang and Bonni, 2016). The catalytic core consists of two subunits, namely the APC2 scaffold protein and the RING domain-containing APC11 (Harper et al., 2002). Cdh1 represents the major regulator and activator of APC in mature neurons and recognizes its substrates via various peptide motifs, including the D (destruction), A and KEN boxes. APC^Cdh1^ plays critical roles in brain development, dendritic integrity, synaptic plasticity, learning and memory (Huang and Bonni, 2016). APC was first shown to regulate the abundance of GLR-1 in *C. elegans*; however no biochemical evidence was presented to suggest a role for APC as the GLR-1 E3 ligase (Juo and Kaplan, 2004). In mammalian neurons, APC^Cdh1^ interacts with the EphA4 receptor to mediate the ephrin-induced proteasomal degradation of AMPARs (Fu et al., 2011). Fu et al. (2011) further demonstrated the ability of APC^Cdh1^ to ubiquitinate GluA1 in HEK293 cells, a process that is dependent on the binding of the Cdh1 WD40 domain to the D box motifs located within the GluA1 extracellular N-terminal domain. Furthermore, mutation of all four lysine residues in the GluA1 C-terminal tail does not inhibit APC^Cdh1^-mediated ubiquitination and degradation in HEK293 cells. Given these facts, we speculate that APC^Cdh1^-mediated ubiquitination of GluA1 may be the result of an overexpression artifact in HEK293 cells, potentially through the activation of the endoplasmic reticulum-associated degradation pathway. It is therefore critical to examine the effect of Cdh1 loss of function on activity-induced AMPAR ubiquitination in neurons. Notwithstanding the clear physiological importance of APC^Cdh1^ in synaptic plasticity, learning and memory, we propose that APC^Cdh1^ may regulate the trafficking of AMPARs through the ubiquitination of AMPAR-related postsynaptic scaffold proteins or other neuronal substrates, including the recently reported Fragile X-associated protein FMRP and the microtubule regulator Rock2 (Huang et al., 2015; Bobo-Jimenéz et al., 2017).

### Deubiquitinating Enzyme for AMPARs

Deubiquitinating enzymes (DUBs) catalyze the removal of covalently attached ubiquitin from proteins, thereby controlling ubiquitin signaling and maintaining the free ubiquitin pool in cells. In contrast to the diversity of ubiquitin E3 ligase, the human genome encodes only ~95 DUBs (Sowa et al., 2009), two of which have been shown to deubiquitinate AMPARs (Scudder et al., 2014; Huo et al., 2015).

#### USP8

The synapse-enriched (DUB), ubiquitin-specific protease 8 (USP8) is a key regulator of the endosomal sorting complex required for transport (ESCRT) pathway that is essential for the formation of multivesicular bodies and lysosomes (Wright et al., 2011; Lee and Gao, 2012). Overexpression of USP8 in cultured neurons reduces the level of agonist-induced AMPAR ubiquitination, leading to increases in the number of surface GluA1 and synaptic AMPARs (Scudder et al., 2014). These effects are abrogated by a mutation of the catalytic cysteine residue in the USP domain, indicating the critical requirement of USP8 deubiquitinating activity in regulating AMPAR trafficking. Furthermore, shRNA-mediated knockdown of USP8 is sufficient to enhance the basal level of AMPAR ubiquitination in primary neurons. The expression and activity of USP8 are regulated by neuronal activity. The activation of NMDARs induces Ca^2+^-dependent dephosphorylation of USP8 by a still unknown tyrosine phosphatase, thereby enhancing its catalytic activity (Scudder et al., 2014).

#### USP46

USP46 was first identified as a DUB that regulates the level of GLR-1 in the ventral nerve cord of *C. elegans* (Kowalski et al., 2011), and was subsequently shown to regulate AMPAR trafficking in mammalian neurons (Huo et al., 2015). USP46 is enriched at synapses and expressed throughout all brain regions. Similar to USP8, knockdown of USP46 also enhances basal GluA1 ubiquitination and reduces its expression in neurons. Congruent with its role as a GluA1 DUB, overexpression of USP46 reduces GluA1 internalization, leading to an increase in the level of surface AMPARs.

### Cross-Talk of AMPAR Phosphorylation and Ubiquitination

In addition to ubiquitination, AMPAR subunits are also subjected to various reversible post-translational modifications, including phosphorylation, palmitoylation and nitrosylation (Roche et al., 1996; Hayashi et al., 2009; Selvakumar et al., 2013). These different post-translational modifications can functionally interact to provide extra layers of fine regulation and modulation of AMPAR trafficking and function (Lu and Roche, 2012). For example, the palmitoylation of the GluA1 subunit at Cys-811 modulates protein kinase C (PKC)-dependent phosphorylation at Ser-816/818, thereby contributing to the regulation of AMPAR insertion into the plasma membrane and synaptic plasticity (Lin et al., 2009). More recently, we demonstrated a cross-talk between GluA1 phosphorylation and ubiquitination. Inhibition of GluA1 ubiquitination causes enhanced protein kinase A (PKA) phosphorylation at Ser-845, but has no effect on PKC/CaMKII phosphorylation at Ser-831 (Guntupalli et al., 2017). Interestingly, the phosphomimetic S845D mutant negatively regulates GluA1 ubiquitination by reducing its ability to interact with Nedd4-1. Such a dynamic cross-modulation of GluA1 ubiquitination and phosphorylation is critical for the membrane sorting of AMPARs, that ultimately determines the number of receptors on the plasma membrane. It remains to be determined whether the ubiquitination of AMPARs causes cross-talk with other post-translational modifications under specific neuronal states.

## Functional Roles of AMPAR Ubiquitination

For historical reasons, ubiquitination has always been associated with protein degradation. However, new roles of ubiquitin in gene transcription, apoptosis, cell cycle, the DNA damage response, cell signaling, protein localization and trafficking have emerged in recent years (Ikeda and Dikic, 2008). The diversity of ubiquitin function is brought about in large part by the ability of ubiquitin to form a polyubiquitin chain that is conjugated on one of its seven lysine residues (Lys-6, Lys-11, Lys-27, Lys-29, Lys-33, Lys-48 and Lys-63) that adopt various structural conformations, thereby creating a range of different molecular signals in the cell (Komander and Rape, 2012). For example, K48-linked polyubiquitination commonly leads proteins towards proteasomal degradation. In contrast, K63-linked substrates often undergo non-proteasomal fates, including protein endocytosis, sorting and receptor trafficking.

### AMPAR Trafficking

The GluA1 and GluA2 subunits of AMPARs are modified by K63-linked polyubiquitin chains when neurons are stimulated with the agonist AMPA in the presence of the NMDAR antagonist, D-APV (Widagdo et al., 2015). It is also well established that the activation of AMPARs in the absence of NMDAR activity induces AMPAR internalization and sorting towards late endosomes for degradation by lysosomes (Ehlers, 2000). Together with the fact that only surface receptors undergo agonist-induced ubiquitination (Widagdo et al., 2015), it is therefore plausible that the ubiquitination of AMPARs is involved in both the endocytosis and post-endocytic sorting of these receptors towards late endosomes. While data from multiple labs have lent support to the role of protein ubiquitination in regulating the intracellular trafficking of AMPARs to late endosomes (Schwarz et al., 2010; Widagdo et al., 2015), the cellular function of GluA1 and GluA2 ubiquitination in mediating AMPAR endocytosis remains controversial (Goo et al., 2015; Widagdo and Anggono, 2015).

There are several pieces of evidence which support the idea that ubiquitination of GluA1 acts as an endocytic signal for AMPARs. These include: (a) overexpression of Nedd4-1 in neurons, which enhances GluA1 ubiquitination, causes a reduction in the number of AMPARs on the plasma membrane (Schwarz et al., 2010; Lin et al., 2011); (b) shRNA-mediated knockdown of Nedd4-1 increases the rate of GluA1 internalization (Schwarz et al., 2010); (c) overexpression of USP46, which downregulates GluA1 ubiquitination, decreases the accumulation of internalized GluA1 in neurons (Huo et al., 2015); and (d) overexpression of GluA1-4KR ubiquitin-deficient mutants (where all four C-terminal lysine residues have been substituted with arginines) blocks AMPA-induced GluA1 internalization (Schwarz et al., 2010; Lin et al., 2011). Moreover, the endocytic adaptor Eps15 (epidermal growth factor receptor substrate 15) has been reported to interact with GluA1 and facilitate AMPAR internalization (Lin and Man, 2014). Interestingly, this interaction is dependent on GluA1 ubiquitination and Eps15 C-terminal ubiquitin interacting motifs. Together, these data support the notion that ubiquitination of AMPARs is necessary for and occurs prior to receptor internalization.

On the other hand, there are two studies that argue against the role of ubiquitin in mediating AMPAR endocytosis (Lussier et al., 2011; Widagdo et al., 2015). In these studies, the authors used small molecule inhibitors of dynamin, an enzyme that is essential for membrane fission during endocytosis, namely dynasore and dynole (Macia et al., 2006; Hill et al., 2009), as well as sucrose to inhibit the formation of clathrin-coated pits, and found that these treatments prevent AMPA- or bicuculline-induced ubiquitination of all AMPAR subunits in cultured neurons. This suggests that endocytosis precedes the conjugation of ubiquitin moieties to AMPARs. Indeed, we found that the same GluA1-4KR ubiquitin-deficient mutant does not affect either the surface expression or agonist-induced internalization of AMPARs (Widagdo et al., 2015), in contrast to the results of previous studies (Schwarz et al., 2010; Lin et al., 2011). Instead, we observed that the GluA1-4KR mutant is mis-sorted into recycling endosomes and returns to the plasma membrane. Interestingly, direct ubiquitination of several transmembrane receptors from the receptor tyrosine kinase and cytokine receptor superfamilies, including the epidermal growth factor receptor, the fibroblast growth factor receptor and the growth factor receptor, is not required for their internalization (Govers et al., 1999; Huang et al., 2007; Haugsten et al., 2008).

The discrepancies found in these studies could arise due to differences in experimental conditions. These may include the duration and intensity of neuronal stimulation, as well as the level of AMPAR subunit overexpression, which could alter the subunit composition of the surface receptors being examined. Given that AMPARs are known to undergo distinct endocytic pathways (Beattie et al., 2000; Ehlers, 2000; Lin et al., 2000), the differential requirement of ubiquitin in regulating the internalization of AMPARs cannot be ruled out. Notwithstanding the debatable role of AMPAR ubiquitination in receptor endocytosis, the consensus is that ubiquitination of AMPARs controls the intracellular sorting of receptors to late endosomes for degradation in lysosomes.

### Synaptic Plasticity

The dynamic trafficking and number of AMPARs on the neuronal plasma membrane are the major determinants of the plasticity of excitatory synapses (Huganir and Nicoll, 2013). The mechanisms that control the endosomal sorting and trafficking of AMPARs are involved in both Hebbian and homeostatic plasticity. For example, protein interacting with C-kinase 1 (PICK1), which directly interacts with GluA2/3 subunits and regulates the endosomal recycling of AMPARs (Xia et al., 1999; Citri et al., 2010; Widagdo et al., 2016), is a critical regulator of long-term potentiation (LTP), long-term depression (LTD) and homeostatic synaptic scaling (Terashima et al., 2008; Thorsen et al., 2010; Anggono et al., 2011). Furthermore, the balance between Rab7- and Rab11-dependent recycling or trafficking of AMPARs towards late endosomes has also been shown to determine the outcome of LTD, underscoring the importance of membrane sorting decisions in synaptic plasticity (Fernández-Monreal et al., 2012). Although there is currently no empirical evidence that demonstrates the role of AMPAR ubiquitination in controlling Hebbian plasticity, there have been several studies that show the involvement of GluA1/2 ubiquitination in homeostatic synaptic scaling (Fu et al., 2011; Hou et al., 2011; Scudder et al., 2014; Jewett et al., 2015).

During prolonged alteration in synaptic activity, homeostatic plasticity maintains neuronal stability by adjusting synaptic properties, including the number of synaptic AMPARs, in order to keep neuronal excitability close to the internal target firing range (Turrigiano, 2012). Downscaling of AMPAR-mediated synaptic currents in primary neurons can be observed during chronic elevation of synaptic activity induced by the blockade of GABA_A_ receptors using bicuculline or picrotoxin (O’Brien et al., 1998; Turrigiano et al., 1998). More recently, homeostatic scaling-down of excitatory synapses has also been observed *in vivo* during sleep, a process that is essential for memory consolidation (Diering et al., 2017).

Recent studies have demonstrated the involvement of Nedd4-1 and Nedd4-2 in mediating homeostatic synaptic downscaling of AMPARs in cultured neurons either by light-controlled single synaptic activation (Hou et al., 2011), or by the application of GABA_A_ receptor antagonists (Scudder et al., 2014; Jewett et al., 2015). Under these conditions, the levels of AMPAR ubiquitination are significantly upregulated concomitant with the reduction of total AMPAR expression in neurons. In one study, Jewett et al. (2015) reported that the transcription of *Nedd4-2* mRNA and Nedd4-2 protein are specifically upregulated following chronic elevation of neuronal activity (Jewett et al., 2015). The detailed mechanisms of picrotoxin-induced increase in Nedd4-2 expression is not well understood, but it involves the Akt-Mdm2-p53 signaling pathway. In contrast, a separate study by Scudder et al. (2014) reported that Nedd4-1 expression could indeed be upregulated following chronic bicuculline treatment. Moreover, they also found that the expression of USP8 was downregulated, further promoting the ubiquitination of AMPARs. Another mechanism that has been shown to control the reduction of AMPAR-mediated synaptic strength involves the APC^Cdh1^-dependent proteasome pathway (Fu et al., 2011). However, the evidence for direct involvement of APC^Cdh1^ in mediating AMPAR ubiquitination and degradation is inconclusive.

## AMPAR Ubiquitination in Neurological Disorders

Homeostatic regulation of the number of ion channels and transmembrane receptors on the plasma membrane is largely achieved by endocytosis mechanisms and downstream endosomal trafficking. Perturbations of AMPAR trafficking have been implicated in a range of neurological disorders. For examples, neuronal hyperactivity due to gain of AMPAR function can lead to epileptic seizures, whereas AMPAR hypofunction is associated with synaptic depression that is commonly linked to disorders such as schizophrenia, chronic stress and Alzheimer’s disease. Recent evidence has started to shed light on the involvement of ubiquitin-mediated trafficking of AMPARs in the pathophysiology of these disorders.

### Alzheimer’s Disease

Early memory deficits and progressive loss of higher cognitive functions are common clinical features of Alzheimer’s disease, which is characterized by the presence of insoluble aggregates of extracellular amyloid-beta (Aβ) peptides and intracellular filaments composed of hyperphosphorylated tau. Strong evidence from human genetics and transgenic mouse models has indicated a role for Aβ in the etiology and pathogenesis of Alzheimer’s disease (Selkoe, 2002). It is well established that soluble oligomeric forms of Aβ peptides exert strong detrimental effects on the structure and functional state of synapses, in part by promoting the internalization and degradation of AMPARs that eventually lead to synaptic depression (Sheng et al., 2012; Guntupalli et al., 2016). In addition to Aβ, a newly identified cleavage product of amyloid precursor protein that is enriched in the dystrophic neurites in an Alzheimer’s disease mouse model and in post-mortem Alzheimer’s brains, termed Aη peptide, has also been shown to impair excitatory synaptic transmission and hippocampal LTP (Willem et al., 2015).

Recent work from our own and the Patrick laboratory have directly demonstrated the direct involvement of AMPAR ubiquitination as a critical pathway in mediating the Aβ-induced synaptic depression in neurons (Rodrigues et al., 2016; Guntupalli et al., 2017). Acute exposure of cultured neurons to soluble Aβ oligomers induces AMPAR ubiquitination concomitant with the removal of the receptors from the plasma membrane (Guntupalli et al., 2017). Importantly, expression of GluA1-K868R or GluA1-4KR ubiquitin-deficient mutants inhibits the adverse effects of Aβ on the surface expression of AMPARs in neurons. Prolonged exposure of neurons to the supernatant of 7PA2 Chinese hamster ovary cells, which naturally secrete Aη peptides (Willem et al., 2015), also enhances AMPAR ubiqutination and consequently causes a reduction in AMPAR-mediated currents and spine loss (Rodrigues et al., 2016). These effects can be rescued by knocking down Nedd4-1 expression, suggesting that the activity of Nedd4-1 is necessary for Aη-induced synaptic alterations in neurons. Given that the expression of Nedd4-1 is upregulated in the human Alzheimer’s brain, a small molecule inhibitor of Nedd4-1 may represent a possible therapeutic for reversing synaptic depression and associated cognitive impairments in patients with this disease.

### Epilepsy

Epilepsy is a chronic neurological disorder characterized by recurring and unprovoked seizures that arise from abnormally synchronous neuronal network activity in a focal area or throughout the entire brain. Genetic or acquired ion channel dysfunctions that alter the excitation and inhibition balance of synaptic connectivity underlie epileptiform discharges. Studies have shown that the inhibition of AMPAR-mediated neuronal excitation confers seizure protection in a broad range of animal seizure models (Rogawski, 2013). Indeed, a highly potent non-competitive AMPAR antagonist, perampanel, has been used clinically to treat patients with partial-onset and tonic-clonic seizures (French et al., 2012, 2015).

Genetic studies have identified at least three different mis-sense mutations in the *Nedd4-2* gene in patients with epilepsy (Dibbens et al., 2007; Epi4K Consortium et al., 2013; Vanli-Yavuz et al., 2015). These mutations disrupt Nedd4-2 binding to 14-3-3ε, thereby reducing its ability to ubiquitinate GluA1 (Zhu et al., 2017). This explains the apparent elevation in seizure susceptibility in the *Nedd4-2*^andi^ mouse, in which the major isoform of Nedd4-2 is selectively deficient in the brain. Remarkably, genetically reducing the GluA1 level by crossing *Nedd4-2*^andi^ mice with *GluA1*^+/−^ heterozygous mice normalizes this effect, underscoring the importance of AMPAR ubiquitination in maintaining the optimal balance of neuronal excitation and inhibition in the brain.

### Chronic Stress

Stress induces the release of glucocorticoids that alter glutamatergic neurotransmission and synaptic plasticity, which could subsequently trigger maladaptive changes in cognitive function (Popoli et al., 2012). It has been shown that repeated stress impairs glutamatergic transmission in the principal neurons in the prefrontal cortex of juvenile male rats (Yuen et al., 2012). One of the underlying mechanisms involves a glucocorticoid receptor-dependent reduction in AMPAR- and NMDAR-mediated synaptic transmission due to enhancement of ubiquitination and proteasomal degradation of GluA1 and GluN1 subunits, respectively. Importantly, shRNA-mediated knockdown of Nedd4-1 and Fbx2 (the E3 ligase for GluN1) in the prefrontal cortex prevents the loss of glutamatergic responses and is able to rescue cognitive deficits in stressed animals. These data further underscore the important roles of AMPAR ubiquitination signaling in mediating synaptic depression under pathological conditions.

## Concluding Remarks

Although studies that emerged in the last few years have demonstrated an important function of the ubiquitin signaling pathway in regulating AMPAR trafficking and function, this field of research is still in its infancy. The roles of AMPAR ubiquitination in controlling receptor endocytosis and degradation (proteasomal vs. lysosomal) are controversial and debatable (Goo et al., 2015; Widagdo and Anggono, 2015), owing to differences in experimental systems, including but not limited to the strength and duration of neuronal stimulation, as well as the model system (primary neurons vs. heterologous cells). Given that AMPARs are not typically present in HEK293 cells, overexpression of any subunit of AMPARs may trigger the cellular stress response and ubiquitin-mediated protein degradation. For this reason, studies of AMPAR ubiquitination in heterologous systems, such as those performed in HEK293 cells, are not ideal and must be interpreted with caution.

**Figure 2:**
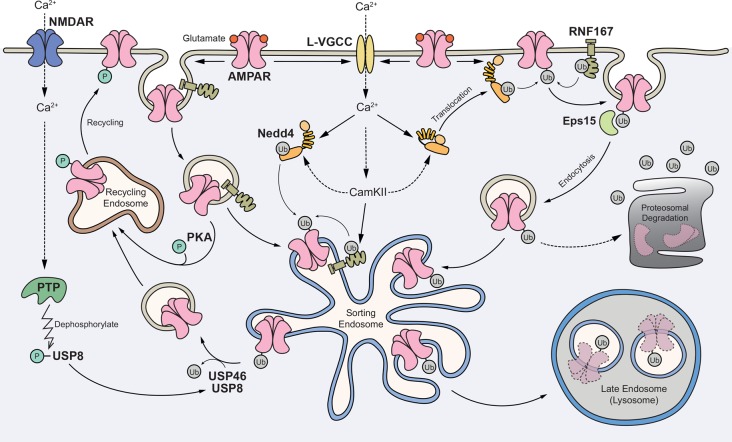
Proposed model for the role of AMPAR ubiquitination in regulating receptor trafficking and degradation. Glutamate binding to AMPARs mediates depolarization of the postsynaptic membrane and the opening of L-type voltage-gated Ca^2+^ channels (L-VGCCs). The rise in intracellular Ca^2+^ subsequently activates E3 ligases through Ca^2+^-dependent translocation of neural precursor cell-expressed developmentally downregulated gene 4-1 (Nedd4-1) to the plasma membrane and/or direct phosphorylation of Nedd4 and RNF167 by Ca^2+^/calmodulin-dependent kinase II (CaMKII). In one scheme, surface AMPARs are internalized without ubiquitination but are subsequently ubiquitinated in the endosomes. In another scheme, ubiquitination of surface receptors recruits the binding of an endocytic adaptor Eps15 and facilitates the internalization of AMPARs. Ubiquitinated AMPARs are sorted to late endosomes and degraded in lysosomes. The activation of NMDARs can recruit USP8, and potentially USP46, to deubiquitinate AMPARs and promote their recycling back to the plasma membrane. Through an unknown mechanism, ubiquitinated AMPARs may also be degraded through the proteasome system.

In summary, we propose the following working model (Figure 2). First, glutamate-mediated activation of AMPARs depolarizes the postsynaptic membrane, leading to the opening of L-VGCCs. The rise in intracellular Ca^2+^ subsequently activates E3 ligases through Ca^2+^-dependent translocation of Nedd4-1 to the plasma membrane and/or direct phosphorylation of Nedd4 and RNF167 by CaMKII. In one scheme, surface AMPARs are internalized without ubiquitination but are subsequently ubiquitinated in endosomes. In another scheme, ubiquitination of surface receptors recruits the binding of an endocytic adaptor, Eps15 and facilitates the internalization of AMPARs. Ubiquitinated AMPARs are then sorted to late endosomes and degraded in lysosomes. The activation of NMDARs can recruit USP8, and potentially USP46, to deubiquitinate AMPARs and promote their recycling back to the plasma membrane. Through an unknown mechanism, ubiquitinated AMPARs may also be degraded through the proteasome system.

Similar to kinases, components of the ubiquitination systems are often dysregulated in disease. Several recent findings have started to implicate dysregulation of AMPAR ubiquitination in the pathophysiology of Alzheimer’s disease, epilepsy and chronic stress (Yuen et al., 2012; Rodrigues et al., 2016; Guntupalli et al., 2017; Zhu et al., 2017). Given the importance of ubiquitination in regulating the fate of receptors, inhibition of E3 ligases and/or DUBs may provide a possible therapeutic mechanism by restoring receptor function (Huang and Dixit, 2016). Future studies should focus on the detailed mechanisms that regulate stimulus- and subunit-specific regulation of AMPAR subunits in neurons during physiological and pathological conditions.

## Author Contributions

JW, SG, SEJ and VA wrote, revised and finalized this manuscript.

## Conflict of Interest Statement

The authors declare that the research was conducted in the absence of any commercial or financial relationships that could be construed as a potential conflict of interest.
